# Pathogenic CD8^+^ T Cells Cause Increased Levels of VEGF-A in Experimental Malaria-Associated Acute Respiratory Distress Syndrome, but Therapeutic VEGFR Inhibition Is Not Effective

**DOI:** 10.3389/fcimb.2017.00416

**Published:** 2017-09-20

**Authors:** Thao-Thy Pham, Melissa Verheijen, Leen Vandermosten, Katrien Deroost, Sofie Knoops, Kathleen Van den Eynde, Louis Boon, Chris J. Janse, Ghislain Opdenakker, Philippe E. Van den Steen

**Affiliations:** ^1^Laboratory of Immunobiology, Department of Microbiology and Immunology, Rega Institute for Medical Research, KU Leuven—University of Leuven Leuven, Belgium; ^2^Translational Cell and Tissue Research, KU Leuven—University of Leuven Leuven, Belgium; ^3^Bioceros Utrecht, Netherlands; ^4^Leiden Malaria Research Group, Department of Parasitology, Leiden University Medical Center Leiden, Netherlands

**Keywords:** PbNK65, mice, lung, VEGF-A, PlGF, malaria

## Abstract

Malaria is a severe disease and kills over 400,000 people each year. Malarial complications are the main cause of death and include cerebral malaria and malaria-associated acute respiratory distress syndrome (MA-ARDS). Despite antimalarial treatment, lethality rates of MA-ARDS are still between 20 and 80%. Patients develop pulmonary edema with hemorrhages and leukocyte extravasation in the lungs. The vascular endothelial growth factor-A (VEGF-A) and the placental growth factor (PlGF) are vascular permeability factors and may be involved in the disruption of the alveolar-capillary membrane, leading to alveolar edema. We demonstrated increased pulmonary VEGF-A and PlGF levels in lungs of mice with experimental MA-ARDS. Depletion of pathogenic CD8^+^ T cells blocked pulmonary edema and abolished the increase of VEGF-A and PlGF. However, neutralization of VEGF receptor-2 (VEGFR-2) with the monoclonal antibody clone DC101 did not decrease pulmonary pathology. The broader spectrum receptor tyrosine kinase inhibitor sunitinib even increased lung pathology. These data suggest that the increase in alveolar VEGF-A and PlGF is not a cause but rather a consequence of the pulmonary pathology in experimental MA-ARDS and that therapeutic inhibition of VEGF receptors is not effective and even contra-indicated.

## Introduction

Malaria is a severe disease which affects 200 million people and causes more than 400,000 deaths each year. Despite the availability of efficient antimalarial treatments and preventive measures, transmission and severe or complicated disease are still reported in 91 countries^1^. Five *Plasmodium* parasites are known to infect the human host: *Plasmodium falciparum, Plasmodium vivax*, *Plasmodium knowlesi, Plasmodium ovale*, and *Plasmodium malariae*. *P. falciparum* is the main species implicated in malarial complications, such as, cerebral malaria (CM) and malaria-associated acute respiratory distress syndrome (MA-ARDS). However, there is an increased incidence of MA-ARDS cases due to *P. vivax* in Southeast Asia and South America (Gupta et al., 2015). This malarial lung pathology is also one of the main complications of malaria caused by *P. knowlesi*, a zoonotic parasite in Southeast Asia (Cox-Singh et al., 2010; William et al., 2011; Van den Steen et al., 2013). MA-ARDS mainly occurs in adults without pre-existing semi-immunity (e.g., travelers and adults in low-transmission areas), has a poor prognosis and leads to a lethality rate of 20–80%, despite antimalarial treatment (Taylor et al., 2012; Van den Steen et al., 2013). Overwhelming pulmonary edema leads to death by impaired gas exchange in the lungs and is paralleled by microhemorrhages and leukocyte infiltration. Currently, the only available therapy is mechanical ventilation (Taylor et al., 2012; Van den Steen et al., 2013).

Vascular endothelial growth factor-A (VEGF-A) is a multipotent molecule that induces survival, migration and proliferation of endothelial cells (ECs) during angiogenesis, but also vascular permeability and endothelial activation during inflammation (Ferrara et al., 2003; Barratt et al., 2014). VEGF-A binds to the tyrosine kinase receptors VEGF receptor-1 (VEGFR-1, also known as flt-1) and VEGF receptor-2 (VEGFR-2, also known as flk-1). Binding of VEGF-A to VEGFR-2 induces signal transduction through tyrosine phosphorylation and results in the induction of angiogenesis, endothelial activation and vascular permeability. In contrast, VEGFR-1 may act as a sink for VEGF-A which prevents its binding to VEGFR-2 (Fischer et al., 2008). PlGF is structurally similar to VEGF-A and only binds to VEGFR-1 (Mura et al., 2004; Takahashi and Shibuya, 2005; Fischer et al., 2008). Similar to VEGF-A, PlGF plays a role in vascular remodeling. Binding of PlGF to VEGFR-1 inhibits the binding of VEGF-A to VEGFR-1 and may therefore increase the availability of VEGF-A to VEGFR-2. However, VEGFR-1 also undergoes weak tyrosine phosphorylation upon VEGF-A and PlGF binding and induces intracellular signal transduction, including transmolecular phosphorylation of VEGFR-2. Additionally, VEGFR-1 can also form a heterodimer with VEGFR-2 (Cudmore et al., 2012). Therefore, PlGF and VEGFR-1 are also able to mediate inflammation and pathological angiogenesis (Fischer et al., 2008).

VEGF-A has been proposed as a malaria severity biomarker in plasma and was also detected in brains of malaria patients, but conflicting data regarding VEGF-A expression levels in malaria have been described (Deininger et al., 2003; Armah et al., 2007; Casals-Pascual et al., 2008; Jain et al., 2008; Yeo et al., 2008; Conroy et al., 2010; Medana et al., 2010; Brouwers et al., 2013; Canavese and Spaccapelo, 2014). VEGF-A has also been detected in lungs of mice with malaria-associated acute lung injury (MA-ALI). Epiphanio et al. developed a mouse model for MA-ALI by infection of DBA/2 mice with *P. berghei* ANKA (PbANKA) (Epiphanio et al., 2010). In this model, serum VEGF-A levels and splenic VEGF-A mRNA expression were increased. Neutralization of the VEGF-A pathway by adenoviral overexpression of the soluble VEGFR-1 (sVEGFR-1) or splenectomy improved survival and therefore the authors proposed a possible role of splenic VEGF-A production in experimental MA-ALI (Epiphanio et al., 2010). Previously, we developed a mouse model for MA-ARDS by infecting C57BL/6 mice with *P. berghei* NK65 (PbNK65). More than 90% of these mice develop fulminant pulmonary pathology (Van den Steen et al., 2010). In this model, alveolar VEGF-A protein levels were increased and correlated with pathology, whereas pulmonary VEGF-A mRNA expression was decreased (Deroost et al., 2013).

In the present study, we further explored the expression and role of VEGF-A in our MA-ARDS model and also included the related PlGF in our analyzes. We observed that the increased levels of both molecules in the bronchoalveolar lavage fluid (BALF) was dependent on the presence of CD8^+^ T cells. Furthermore, we evaluated whether therapeutic inhibition with DC101, a monoclonal antibody against VEGFR-2, or with sunitinib, a small molecule inhibitor of tyrosine kinase receptors with a broader spectrum than DC101, is able to inhibit pulmonary pathology and to improve survival in this preclinical model. However, no beneficial effects of these inhibitors could be observed, and our data indicated the increase of VEGF-A and PlGF in the lungs to be a consequence rather than a cause of MA-ARDS.

## Materials and methods

### Ethical statement

All experiments were approved by the Animal Ethics Committee from the KU Leuven (License LA1210186 Belgium). Experiments for the generation of the PbNK65 2168cl2 line were approved by the Animal Experiments Committee of the Leiden University Medical Center (DEC 12042).

### Mice and parasites

Unless otherwise indicated, male and female C57BL/6 mice were obtained from Janvier (7–8 weeks old, Le Genest-Saint-Isle, France). Mice were infected with PbNK65 or an isogenic clone of PbNK65, PbNK65 2168cl2 (see below for the generation of this clone) by intraperitoneal (IP) injection of 10^4^ infected red blood cells (Van den Steen et al., 2010). Mice were kept in a conventional animal house and drinking water was supplemented with 4-amino benzoic acid (0.375 mg/ml, PABA, Sigma-Aldrich, Bornem, Belgium). Parasitemia was determined by microscopic analysis of blood smears of tail blood after Giemsa staining (1/10 dilution, VWR, Heverlee, Belgium). Mice were sacrificed at indicated time points after infection by euthanasia with Dolethal (Vétoquinol, Aartselaar, Belgium; 200 mg/ml, IP injection of 50 μl).

### Quantification of lung pathology and VEGF-A and PlGF protein levels

Lung pathology was assessed by measuring the protein concentration in BALF. To obtain BALF, 750 μl PBS was instilled in both lungs through the trachea with a catheter and withdrawn after 30 s. This was repeated and both lavages were pooled. The BALF was centrifuged (10 min at 335 g, 4°C) and the protein concentration of the supernatant was determined by Bradford assay (Bio-Rad, Hercules, CA, USA). Alveolar and plasma VEGF-A and PlGF concentrations were determined in BALF with a Duoset ELISA kit, according to the manufacturer's instructions (R&D Systems Europe Ltd., Abingdon, UK).

### Quantitative reverse transcription-polymerase chain reaction

After mechanical homogenization of the left lungs, total RNA was extracted (RNeasy mini kit, Qiagen, Hilden, Germany) and quantified (Nanodrop, Thermo Fischer, Aalst, Belgium), cDNA was synthesized (High capacity cDNA reverse transcription kit, Thermo Fischer), and quantitative reverse transcription-polymerase chain reaction (qRT-PCR) was performed on 25 and 12.5 ng cDNA with primer and probe sets from Integrated DNA Technologies (Leuven, Belgium). Data were normalized to the uninfected controls (CON) and 18S ribosomal RNA determinations (Livak and Schmittgen, 2001). *P. berghei* (Pb) 18S was used to determine pulmonary parasite accumulation (Penha-Gonçalves Costa et al., 2013). Pb 18S data was normalized to the corresponding expression of the murine 18S RNA.

### Immunohistochemistry

During dissection, 750 μl formalin (4%) was instilled in the lungs with a catheter. Thereafter, lungs were completely submerged in formalin (4%) for 24 h. Paraffin sections were pre-treated with citrate (pH 6). Subsequently, endogenous peroxidase activity of the lung sections were blocked for 5 min with a peroxide block (Bond Polymer Refine Detection kit, Leica, Diegem, Belgium). The sections were further incubated for 30 min at room temperature (RT) with monoclonal rabbit anti-mouse VEGF-A (dilution 1/100; EP1176Y, Abcam, Cambridge, UK) in Bond Primary Antibody Diluent (Leica), HRP-labeled goat-anti rabbit (8 min at RT), 3,3′-diaminobenzidine tetrahydrochloride hydrate (DAB) chromogen (10 min, Bond Polymer Refine Detection kit, Leica), and hematoxyline (5 min, Bond Polymer Refine Detection kit, Leica), using an autostainer (Leica). Transmitted light images were taken through a 10x/0.25 or a 40x/0.65 N Plan objective of a Leica DM2000 microscope. Image background adjustments were performed with the AxioVision 4.6 software (Zeiss, Zaventem, Belgium).

### *In vivo* depletion of CD8^+^ T cells

PbNK65-infected mice were treated with 0.5 mg monoclonal anti-CD8β (rat IgG2b hybridoma clone H35-17-2, kindly provided by Prof. P. Matthys, Rega Institute for Medical Research, KU Leuven - University of Leuven, Leuven, Belgium) dissolved in 200 μl PBS by IP injection on day 7 *post-*infection (pi) as described in Van den Steen et al. (Van den Steen et al., 2010). As controls, PbNK65-infected mice were injected IP with 200 μl PBS.

### *In vivo* VEGFR-2 neutralization or sunitinib treatment

Monoclonal rat anti-VEGFR-2 antibody (clone DC101, Bio X Cell, NH, USA) or isotype antibody IgG1a [Anti-β-Gal, GL113, kind gift of Dr. Louis Boon (Bioceros, Utrecht, The Netherlands)] was used for the *in vivo* neutralization experiments. Mice were injected IP on day 6 pi with 900 μg anti-VEGFR-2 or IgG1a (Oladipupo et al., 2011). Sunitinib (sunitinib malate, Bioconnect, Huissen, The Netherlands) was dissolved at 10 mg/ml in 80 mM citrate pH 2.5. From day 6 pi on, mice were gavaged daily with 100 μl sunitinib solution or with the vehicle (Payen et al., 2014).

### Generation of PbNK65 2168cl2

For the generation of the PbNK65 2168cl2 cloned line, Swiss OF1 mice (6 weeks old; 25–26 g; Charles River, Leiden, The Netherlands) and the reference GIMO (“gene insertion/marker out”) 1995cl2 mother cloned line (Lin et al., 2014) of the PbNK65 parental line (Van den Steen et al., 2010) were used (Supplementary Figure 1). This 1995cl2 mother cloned line contains a fusion of a positive drug-selectable marker (*hdhfr*) and a negative drug-selectable marker (*yfcu*) integrated into the neutral *230p* locus, which allows rapid introduction of transgenes into the PbNK65 genome without drug-resistance markers, using the standard methods of GIMO transfection (Lin et al., 2011). Parasites of the PbNK65 GIMO 1995cl2 mother cloned line were transfected with construct pL1156 that contains a GFP-luciferase expression cassette and *230p* targeting sequences (Supplementary Figure 1). The *gfp-luciferase* gene is under control of the schizont-specific *ama1* promoter. After transfection, negative selection was applied to select for parasites in which the h*dhfr*::y*fcu* selection cassette in the *230p* locus is replaced by the expression cassette. The selected parasites (2168 cloned lines) were cloned by the method of limiting dilution and the 2168cl2 cloned line was genotyped by Southern blot analysis of pulsed field gel electrophoresis (PFGE) separated chromosomes (Janse et al., 2006).

### Statistical analysis

The Mann-Whitney U test was used to determine the statistical significance. Statistical analysis was done using the GraphPad Prism software (GraphPad software, San Diego, USA). *P*-values smaller than 0.05 were considered statistically significant. *P*-values were defined as followed ^*^p < 0.05, ^**^p < 0.01, ^***^p < 0.001, ^****^p < 0.0001. Occasional mice in which the parasite did not develop well (<1% parasitemia on day 7 pi for PbNK65 2168cl2 and on day 8 pi for PbNK65) were excluded. Unless otherwise specified, each dot represents the results from an individual mouse. Horizontal lines represent group medians. Asterisks without horizontal lines represent significant differences compared to the uninfected control group (CON). Horizontal lines with asterisk on top indicate pairwise significant differences between cohorts of animals.

## Results

### Vascular permeability factors are induced during experimental MA-ARDS

**Figure 1:**
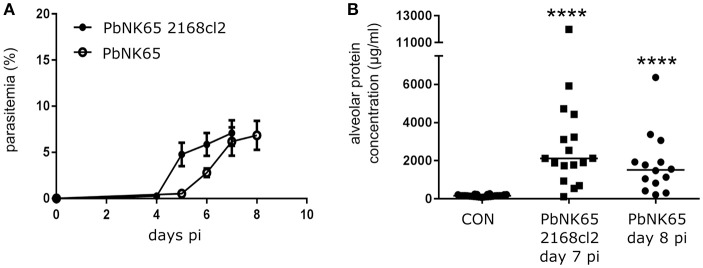
PbNK65 2168cl2 and PbNK65-infected C57BL/6 mice develop MA-ARDS. C57BL/6 mice were infected with PbNK65 2168cl2 or PbNK65. **(A)** Peripheral parasitemia was determined at indicated days with Giemsa-stained smears of tail blood. Mean ± SEM are indicated. Compilation of two experiments, *n* = 10–20 per group. **(B)** Alveolar edema was measured by protein determination of BALF samples, collected at day 7 and day 8 pi from mice infected with PbNK65 2168cl2 or PbNK65, respectively. After centrifugation, the protein content of the supernatant was determined. Compilation of three experiments, *n* = 14–26 per group.

**Figure 2:**
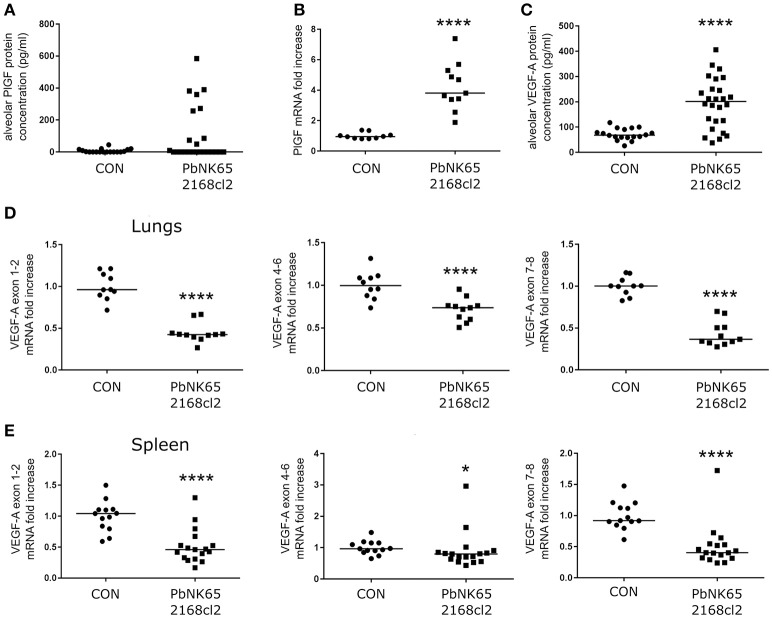
PlGF and VEGF-A are increased in lungs of mice with MA-ARDS. Mice infected with PbNK65 2168cl2 were dissected at day 7 pi. **(A)** Protein levels of PlGF were determined in BALF. **(B)** The mRNA expression levels of PlGF were measured in total lung extracts. **(C)** Protein levels of VEGF-A were determined in BALF. **(D,E)** The mRNA levels of different splice variants of VEGF-A were determined in extracts of lungs and spleen. **(B,D)** Two experiments, **(E)** three experiments, **(A)** four experiments, and **(C)** five experiments, *n* = 10–27 per group.

VEGF-A is an important vascular permeability factor that increases endothelial permeability *in vitro* (Gavard and Gutkind, 2006). In addition, overexpression of sVEGFR-1 with an adenoviral vector inhibits MA-ALI in PbANKA-infected DBA/2 mice, suggesting that VEGF-A or PlGF may play a pathogenic role (Epiphanio et al., 2010). Therefore, we investigated the expression of VEGF-A and PlGF in our experimental MA-ARDS model with C57BL/6 mice infected with PbNK65. Hereby, we used two different parasite lines, the parental PbNK65 line (Van den Steen et al., 2010) and an isogenic cloned line derived from this parental line, PbNK65 2168cl2. This cloned line had a slightly increased growth rate compared to the parental PbNK65 line, but was otherwise similar and caused lethal MA-ARDS without neurological symptoms at 7–8 days pi (Figure 1). As demonstrated further, the results with both parasites confirm each other. With PbNK65 2168cl2, the lung pathology was accompanied by a trend toward increased PlGF protein levels in BALF and a significant increase of pulmonary PlGF mRNA expression (Figures 2A,B). Alveolar VEGF-A protein was also increased on day 7 pi (Figure 2C), but not yet on day 6 pi (Supplementary Figure 2B), suggesting that the increase occurred at end-stage disease.

The gene encoding VEGF-A comprises eight exons both in mice and humans. Due to alternative splicing of these exons, VEGF-A variant isoforms are created (Tischer et al., 1991). In contrast to alveolar VEGF-A protein level, none of the VEGF-A isoform mRNAs were upregulated in the lungs as indicated by qRT-PCR of lung extracts (Figure 2D). Epiphanio et al. described an increase in VEGF-A mRNA in the spleen of PbANKA-infected DBA/2 mice with MA-ALI/ARDS (Epiphanio et al., 2010). However, qRT-PCR analysis of spleen extracts from our PbNK65 2168cl2-infected C57BL/6 mice did not show any mRNA increase of the different VEGF-A isoforms (Figure 2E).

**Figure 3:**
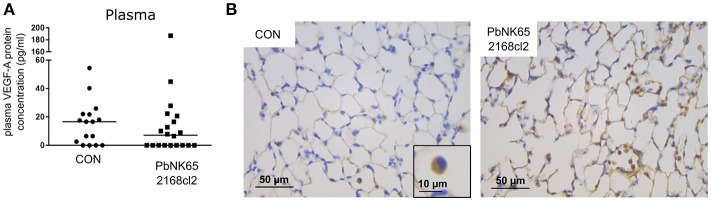
Plasma levels and pulmonary immunohistochemical detection of VEGF-A in murine MA-ARDS. C57BL/6 mice were infected with PbNK65 2168cl2. **(A)** Plasma levels of VEGF-A were determined at day 7 pi. Compilation of four experiments, *n* = 16–20. **(B)** Paraffin sections were prepared from lungs of uninfected (CON) and infected mice, and stained with anti-VEGF-A monoclonal antibody (brown). Representative images are shown in 40x/0.65 magnification (scale bars, 10 mm), *n* = 2–4.

The VEGF-A detected in BALF samples did not originate from plasma leaking into the alveoli, since no increases in plasma levels of VEGF-A were detected (Figure 3A). In addition, VEGF-A protein levels in the plasma were also in a lower range compared to BALF. We also performed immunohistochemistry of lung sections, which were stained with anti-VEGF-A monoclonal antibody. Consistent with the known constitutive expression of VEGF-A in lungs (Barratt et al., 2014), VEGF-A staining was seen in alveolar macrophages and alveolar septa in lungs of uninfected C57BL/6 mice (Figure 3B). Stronger immunoreactive staining was observed in alveolar septa of infected mice, compared to the uninfected mice. These data confirm increased VEGF-A protein levels in lungs of infected mice.

### Depletion of CD8^+^ T cells decreases pulmonary VEGF-A and PlGF levels

**Figure 4:**
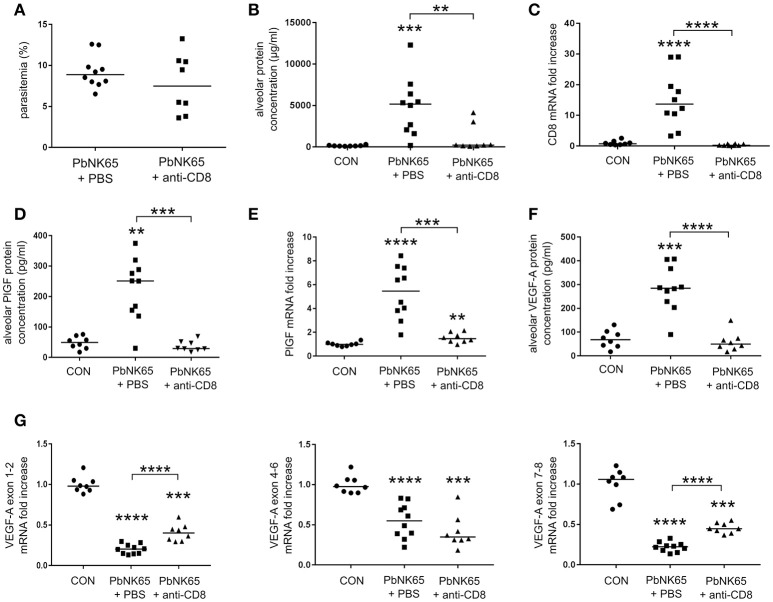
Alveolar edema and expression of VEGF-A and PlGF are dependent on pathogenic CD8^+^ T cells. C57BL/6 mice were infected with PbNK65 and treated with anti-CD8β antibodies to deplete CD8^+^ T cells. The effect of the depletion on **(A)** peripheral parasitemia and **(B)** alveolar edema was determined. **(C)** Quantitative RT-PCR for CD8 indicated the efficacy of the depletion. **(D,F)** The alveolar protein levels of PlGF and VEGF-A and **(E,G)** pulmonary mRNA expression of PlGF and of the different VEGF-A isoforms were compared between CD8^+^ T-cell depleted and mock-treated animals. Compilation of two experiments, *n* = 8–10 per group.

Previously, we have shown that CD8^+^ T cells play an important role in the development of MA-ARDS, since depletion of CD8^+^ T cells diminished the MA-ARDS-associated increase in lung weight (Van den Steen et al., 2010). Using the PbNK65 parental line, we confirmed that CD8^+^ T cell depletion almost completely abolished alveolar edema without affecting parasitemia (Figures 4A–C). CD8^+^ T cell depletion impaired the increases in the alveolar PlGF and VEGF-A protein levels (Figures 4D,F). In addition, PlGF mRNA expression decreased after CD8^+^ T cell depletion, whereas VEGF-A isoform mRNA expression levels remained unchanged (Figures 4E,G). These data indicate that the increases in VEGF-A and PlGF protein levels are dependent on the presence of CD8^+^ T cells. Furthermore, we confirmed that the increases in PlGF and VEGF-A occur only at end-stage disease both in mice infected with the PbNK65 2168cl2 cloned line and in mice infected with the PbNK65 parental line (Figures 2, 4 and Supplementary Figures 2, 3).

### Neutralization of VEGFR-2 with the anti-VEGFR-2 antibody clone DC101 does not decrease lung pathology

**Figure 5:**
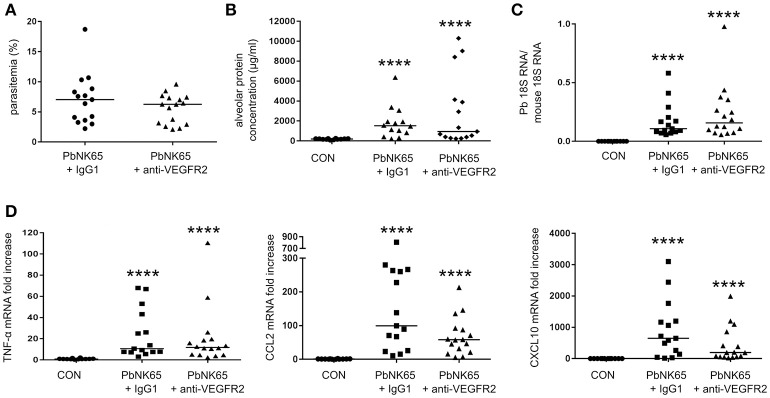
Treatment of PbNK65-infected mice with anti-VEGFR-2 Ab does not decrease lung pathology. C57BL/6 mice were infected with PbNK65 and were treated by IP injection of the VEGFR-2-neutralizing antibody clone DC101 (anti-VEGFR-2) or with an isotype control (IgG1a) at day 6 pi. **(A)** Peripheral parasitemia at day 8 pi is shown and **(B)** alveolar edema was measured by protein determination in BALF samples. **(C,D)** The mRNA expression of *P. berghei* 18S, TNF-α, CCL2, and CXCL10 was analyzed by qRT-PCR. Compilation of three experiments, *n* = 12–16 per group.

To investigate whether the VEGF-A pathway could be a therapeutic target in MA-ARDS, we neutralized VEGFR-2 with a monoclonal anti-VEGFR-2 antibody clone DC101. Treatment of PbNK65-infected mice did not significantly affect parasite growth, pulmonary parasite accumulation, alveolar edema, and pulmonary mRNA expression of TNF-α, CCL2, and CXCL10 (Figure 5). These results indicated that therapeutic inhibition of VEGFR-2 is not effective to block the pulmonary pathology in our experimental MA-ARDS model.

### Inhibition of VEGFR-1 and VEGFR-2 with sunitinib increases lung pathology

**Figure 6:**
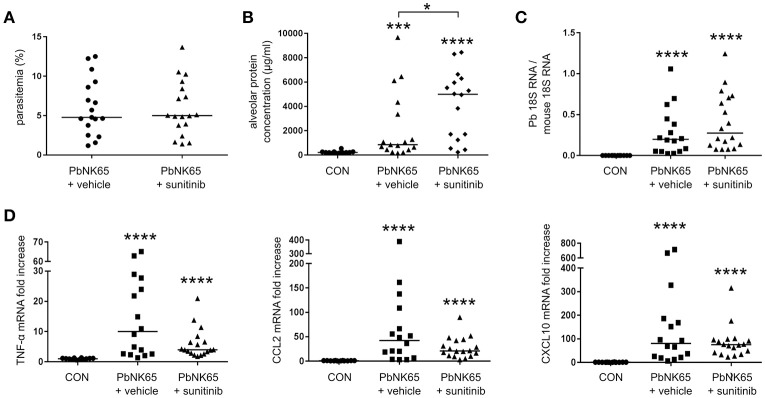
Treatment of mice with sunitinib increases lung pathology in PbNK65-infected C57BL/6 mice. C57BL/6 mice were infected with PbNK65 and were treated by daily gavage from day 6 pi on with sunitinib or with vehicle. **(A)** Peripheral parasitemia was determined at day 8 pi and **(B)** alveolar edema was measured by protein determination of BALF samples. **(C,D)** The mRNA expression of *P. berghei* 18S, TNF-α, CCL2, and CXCL10 was analyzed by qRT-PCR. Compilation of three experiments, *n* = 12–18 per group.

Since no difference in pathology was seen with the anti-VEGFR-2 antibody, we treated infected mice with a broader spectrum VEGF-A inhibitor, the small molecule tyrosine kinase inhibitor sunitinib. Sunitinib binds and blocks receptor tyrosine kinases, including VEGFR-1 and VEGFR-2 (Takahashi, 2011). Surprisingly, sunitinib administration increased lung pathology in PbNK65-infected mice (Figure 6). This was not the result of an effect of sunitinib on the course on infection, since the parasitemia and pulmonary parasite accumulation were similar at day 8 pi in treated and untreated mice (Figures 6A,C). Sunitinib also had no significant outcome on pulmonary mRNA expression of TNF-α, CCL2, and CXCL10 (Figure 6D). These data indicated that inhibition of receptor tyrosine kinases with sunitinib is not protective and rather aggravates the pathology.

## Discussion

VEGF-A is a multifunctional molecule, as it induces EC survival and activation, vascular permeability and vascular remodeling (Barratt et al., 2014). Mura et al. proposed a damaging role of VEGF-A in the early onset of non-malarial ARDS. This is followed by a decrease in VEGF-A levels due to neutrophil-induced degradation by neutrophil proteases and apoptosis of lung epithelial cells (Mura et al., 2004). A subsequent protective increase of alveolar VEGF-A may help to restore the alveolar-capillary membrane. The pathogenesis of MA-ARDS is, however, different from classical ARDS. Besides a limited increase in neutrophils, abundant accumulation of CD8^+^ T cells and monocytes/macrophages are observed in the lungs, and the CD8^+^ T cells play a crucial pathogenic role (Van den Steen et al., 2013; Lagassé et al., 2016; Sercundes et al., 2016). In our experimental MA-ARDS model, an increase of alveolar VEGF-A is only observed at end stage disease.

This sudden increase of VEGF-A protein levels did not coincide with VEGF-A mRNA expression in the lungs (Figures 2C,D). Also at earlier time points, the VEGF-A mRNA was not increased (Supplementary Figure 2). It has been hypothesized that VEGF-A-containing macrophages might migrate from the spleen to the lungs to locally secrete VEGF-A (Epiphanio et al., 2010). However, in our experimental MA-ARDS model no increase of VEGF-A mRNA expression was observed in the spleen (Figure 2E). Ekekezie et al. also observed decreased pulmonary VEGF-A mRNA and increased VEGF-A protein levels in BALF of hyperoxic piglets (Ekekezie et al., 2003). They suggested that due to hyperoxia, activated proteases cleave the VEGF-A from the extracellular matrix and release the molecule into the alveolar space. However, this was not the case in our MA-ARDS model, since we observed an increased VEGF-A protein expression by immunohistochemistry of the lungs in our PbNK65-infected mice compared to the control, which suggested enhanced production of the VEGF-A protein in absence of an overall increase in VEGF-A mRNA (Figure 3B).

In a model of MA-ALI with PbANKA infected-DBA/2 mice, overexpression of sVEGFR-1 was shown to decrease the pulmonary pathology (Epiphanio et al., 2010). However, our data demonstrated that therapeutic inhibition of VEGFR-1 and VEGFR-2 with sunitinib, a small molecule broad-spectrum receptor tyrosine kinase inhibitor, resulted in more pathology in experimental MA-ARDS, whereas specific inhibition of VEGFR-2 had no effect (Supplementary Figure 4). The difference between our results and those from Epiphanio et al. may be due to the different mouse models used (PbANKA-infected DBA/2 vs. PbNK65-infected C57BL/6 mice) and/or to the method used to inhibit the VEGF-A pathway (sVEGFR-1 overexpression vs. therapeutic inhibition with a VEGFR-2 blocking antibody or with sunitinib).

In human lungs, PlGF is expressed by alveolar type II epithelial cells and macrophages (Janér et al., 2008). A correlation between alveolar PlGF and pulmonary pathology was found in rats with hyperoxia-induced ALI (Zhang et al., 2015). PlGF also induces focal adhesion disassembly and apoptosis *in vitro* in murine alveolar type II epithelial cells, suggesting a role of alveolar PlGF in the disruption of the alveolar capillary barrier (Tsao et al., 2004; Zhang et al., 2015, 2016a). In fact, the inhibition of PlGF expression with lentivirus particles containing PlGF-specific shRNA impairs hyperoxia-induced lung pathology (Zhang et al., 2016a,b). These studies indicate that PlGF may be pathogenic in the lungs by inducing vascular permeability. Until now, the role of PlGF was not described in malaria. We observed an increase of PlGF protein concentration and mRNA expression in lungs of PbNK65-infected mice.

Since both VEGF-A, PlGF and their receptors induce angiogenesis, monoclonal antibodies against these molecules have been used as a therapy for cancer (Fischer et al., 2008). For example, the humanized monoclonal antibody against VEGF-A bevacizumab is used to inhibit angiogenesis. However, patients develop severe side effects, as it influences the maintenance of ECs (Verheul and Pinedo, 2007). Moreover, resistance against anti-VEGF-A occurs due to redundancy of other angiogenic factors. Despite these problems, anti-VEGF-A is now used in clinical trials and is shown to be effective when combined with chemotherapy (Hurwitz et al., 2004). Anti-PlGF therapy induces less adverse effects than anti-VEGF-A treatment and has currently reached phase 1 and phase 2 clinical trials^2^. Additionally, both sVEGFR-1 (“VEGF trap”) and anti-VEGFR-2 (ramucirumab) have entered phase 1 clinical trials^2^. Small molecules such as, sunitinib, a receptor tyrosine kinase inhibitor, are also used for anti-angiogenic therapy. We observed that sunitinib aggravated the lung pathology in our PbNK65-infected mice, leading to more severe edema formation. Despite the side effects, therapies that inhibit the VEGF-A pathway are used to treat cancers, as their efficacy trumps the “collateral damage.” However, in MA-ARDS there is no benefit. We observed no amelioration of pathology by blocking VEGFR-2 and even demonstrated an increase in severity with sunitinib treatment in our model of experimental MA-ARDS.

Our results also indicated that the increase of VEGF-A and PlGF in the lungs are dependent on the presence of CD8^+^ T cells. CD8^+^ T cells are known to play an important role in experimental cerebral malaria (ECM) by migrating to the brains and by exerting cytotoxic activities through granzyme B and perforin (Howland et al., 2015a). Locally released granzyme B and perforin damage the ECs of the blood brain barrier, which eventually results in cerebral edema. The chemokine receptor CXCR3 and its ligands CXCL9 and CXCL10 are responsible for the chemoattraction of activated T cells from the spleen to the brain in ECM (Campanella et al., 2008; Miu et al., 2008; Van den Steen et al., 2008; Nie et al., 2009). CD8^+^ T cells in brains of PbANKA-infected mice showed specificity for several parasite-derived antigens (Howland et al., 2015a). Howland et al. demonstrated cross-presentation of malarial antigens by brain vessels in PbANKA-infected mice (Howland et al., 2015b). Transfer of *P. falciparum* parasite antigens to human brain ECs was also observed *in vitro* (Jambou et al., 2010). Parasite antigen-specific CD8^+^ T cells may therefore target the brain ECs and further aggravate the damage to the blood brain barrier. Similar to these observations in ECM, depletion of CD8^+^ T cells in our experimental MA-ARDS model almost completely inhibited pathology (Figure 4B; Van den Steen et al., 2010). How these CD8^+^ T cells exactly play a role in the MA-ARDS pathogenesis is still under investigation, but the expression of CXCL10 in the lungs and CXCR3 on the CD8^+^ T cells suggest that these cytotoxic cells are recruited to the lungs in experimental MA-ARDS as in ECM (Van den Steen et al., 2010; Deroost et al., 2013). Our results indicated that pathogenic CD8^+^ T cells induced experimental MA-ARDS and thereby upregulated VEGF-A and PlGF protein levels in the lungs. Since inhibition of the VEGF-A/PlGF pathway had no therapeutic effect, it is likely that the VEGF-A and PlGF increases are rather a consequence of the MA-ARDS pathology and not a cause, as summarized in Supplementary Figure 4.

## Author contributions

TP performed all experiments with PbNK65 2168Cl2 and the experiments with the VEGFR2 neutralizing antibody or sunitinib. TP wrote the manuscript. MV executed the CD8 depletion experiment, under supervision of TP. LV assisted in the VEGFRs neutralization experiments. KD participated in the experiments with PbNK65 2168Cl2 together with TP. SK did the ELISAs of all experiments. KVdE optimized and executed the histological analyzes. LB delivered the isotype antibody IgG1a. CJ generated the 2168cl2 parasite. GO contributed to the revision of the manuscript and the supervision of the project. PV contributed to the writing and critical reading of the manuscript and supervised the project. All authors read and approved the final manuscript.

### Conflict of interest statement

The authors declare that the research was conducted in the absence of any commercial or financial relationships that could be construed as a potential conflict of interest.

## Footnotes

^1^WHO world malaria report. http://www.who.int/malaria/publications/world_malaria_report

^2^*Clincal Trials*. Available online at: http://www.clinicaltrials.gov

## Supplementary material

Supplementary Figure 1 Generation and analysis of the PbNK65 2168cl2 parasite. **(A)** Schematic representation showing the introduction of GFP-luciferase expression cassette into the reference PbNK65 GIMO cloned line, GIMO_NK65_ (1995cl2). In construct pL1156 the fusion gene *gfp::luciferase* is under control of the schizont specific *ama1* promoter. The construct integrates into the modified *230p* locus of GIMO_*NK*65_containing the *hdhfr::yfcu* selectable marker cassette by double cross-over homologous recombination at the target regions (gray boxes). Negative selection with 5-fluorocytosine selects for the transgenic parasites (2168) that have the GFP-luciferase-expression cassette introduced into the *230p* locus and the *hdhfr::yfcu* marker removed. **(B)** Southern blot analysis of separated chromosomes confirms the correct integration of construct pL1156 into the *P. berghei* genome of the PbNK65 2168cl2 cloned line. Chromosomes were hybridized using a mixture of two probes: (i) the *hdhfr* probe that recognizes the *hdhfr::yfcu* selectable marker cassette in the GIMO_NK65_ cloned line in the *230p* locus on chromosome 3 and (ii) a control probe (p25) that recognizes chromosome 5 (Lin et al., 2011).

Supplementary Figure 2 Limited lung pathology on day 6 pi in PbNK65 2168cl2-infected C57BL/6 mice. C57BL/6 mice were infected with PbNK65 2168cl2. **(A)** BALF samples were collected at day 6 pi. After centrifugation, the protein content of the supernatant was determined. **(B)** The VEGF-A protein content of BALF was measured. **(C)** Lungs were dissected at indicated days pi. Lungs were further homogenized and the mRNA expression of a VEGF-A isoform (primer VEGF-A exon 4-6) was analyzed by qRT-PCR. **(D)** The PlGF protein content of BALF, and **(E)** mRNA expression of PlGF were determined. Compilation of 2 experiments, *n* = 2–11 per group.

Supplementary Figure 3 Pulmonary VEGF-A and PlGF protein concentrations during PbNK65 infection in C57BL/6 mice. C57BL/6 mice were infected with PbNK65. **(A)** Peripheral parasitemia was determined at indicated days with Giemsa-stained smears of tail blood and **(B)** alveolar edema was measured by protein determination in BALF samples. Protein levels of **(C,D)** VEGF-A and PlGF were also determined in BALF. Compilation of three experiments, *n* = 7–18 per group.

Supplementary Figure 4 Schematic overview of anti-VEGFR-2 and sunitinib treatment in infected mouse lungs. This figure depicts a scheme of the interrelations of the VEGF-A and PlGF pathway with the anti-VEGFR-2 antibody (DC101 clone) and sunitinib treatment in infected mouse lungs. The target cells may be ECs, alveolar type II cells and macrophages. CD8^+^ T cells were shown to be essential for lung pathology. A downstream effect of this CD8^+^ T cell-mediated lung pathology is the expression of VEGF-A and PlGF in the lungs, whose receptors are VEGFR-1 and VEGFR-2. The anti-VEGFR-2 antibody inhibits the binding of VEGF-A to VEGFR-2, resulting in decreased VEGFR-2 activation. Sunitinib impairs the tyrosine kinase activity of VEGFR-1 and VEGFR-2 which leads to decreased activation of both receptor tyrosine kinases. However, both treatments did not impair CD8^+^ T cell-mediated lung pathology and sunitinib even aggravated the pathology.
