# Supplementary figures and images for: Pathogenic CD8+ T Cells Cause Increased Levels of VEGF-A in Experimental Malaria-Associated Acute Respiratory Distress Syndrome, but Therapeutic VEGFR Inhibition Is Not Effective
Source: Front Cell Infect Microbiol. 2017 Sep 20;7:416. doi: 10.3389/fcimb.2017.00416 (PMC5627041; doi:10.3389/fcimb.2017.00416)

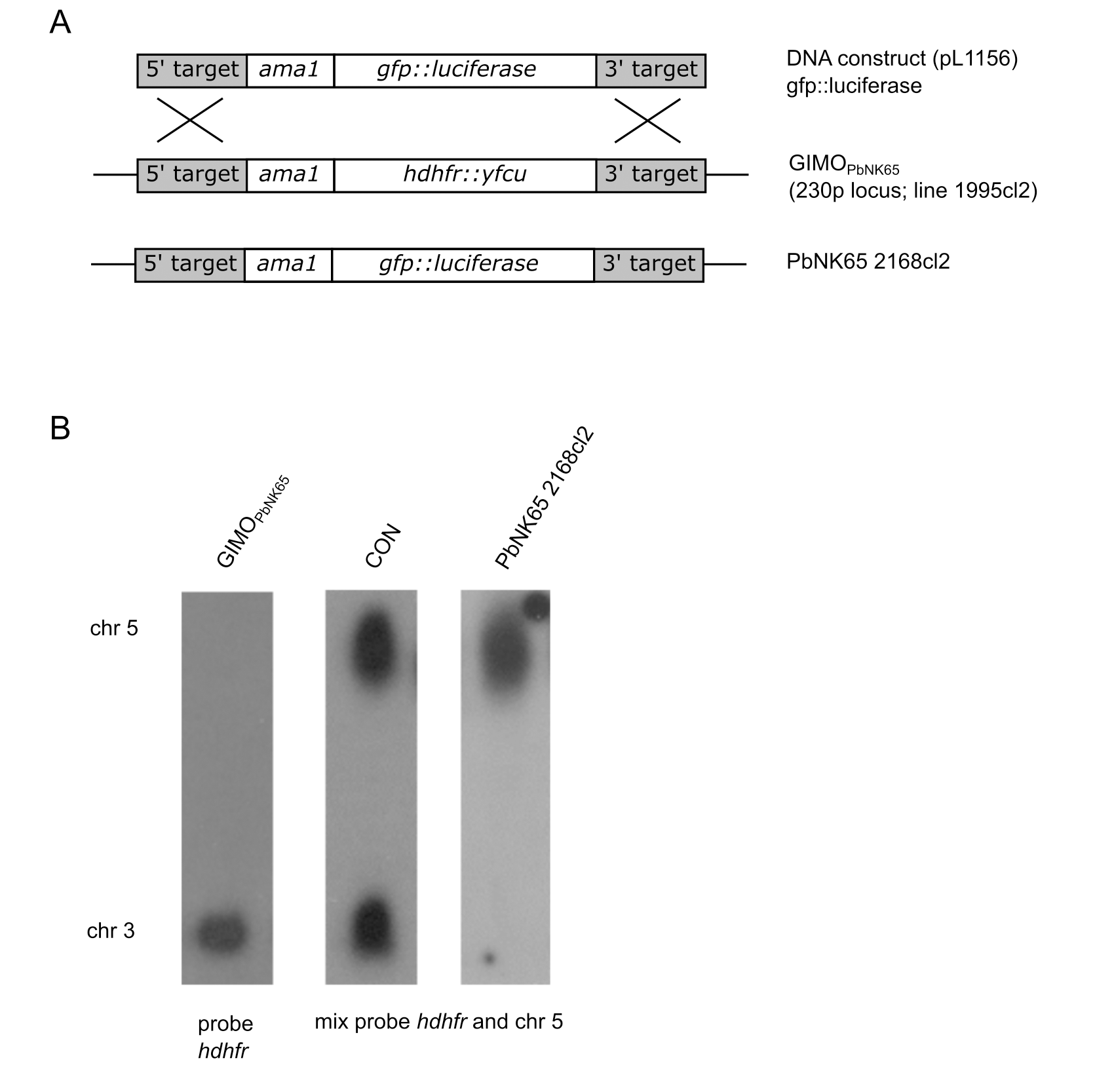

Supplement: Supplementary Figure 1 — Generation and analysis of the PbNK65 2168cl2 parasite. (A) Schematic representation showing the introduction of GFP-luciferase expression cassette into the reference PbNK65 GIMO cloned line, GIMONK65 (1995cl2). In construct pL1156 the fusion gene gfp::luciferase is under control of the schizont specific ama1 promoter. The construct integrates into the modified 230p locus of GIMONK65containing the hdhfr::yfcu selectable marker cassette by double cross-over homologous recombination at the target regions (gray boxes). Negative selection with 5-fluorocytosine selects for the transgenic parasites (2168) that have the GFP-luciferase-expression cassette introduced into the 230p locus and the hdhfr::yfcu marker removed. (B) Southern blot analysis of separated chromosomes confirms the correct integration of construct pL1156 into the P. berghei genome of the PbNK65 2168cl2 cloned line. Chromosomes were hybridized using a mixture of two probes: (i) the hdhfr probe that recognizes the hdhfr::yfcu selectable marker cassette in the GIMONK65 cloned line in the 230p locus on chromosome 3 and (ii) a control probe (p25) that recognizes chromosome 5 (Lin et al., 2011). [file Image1.TIF]

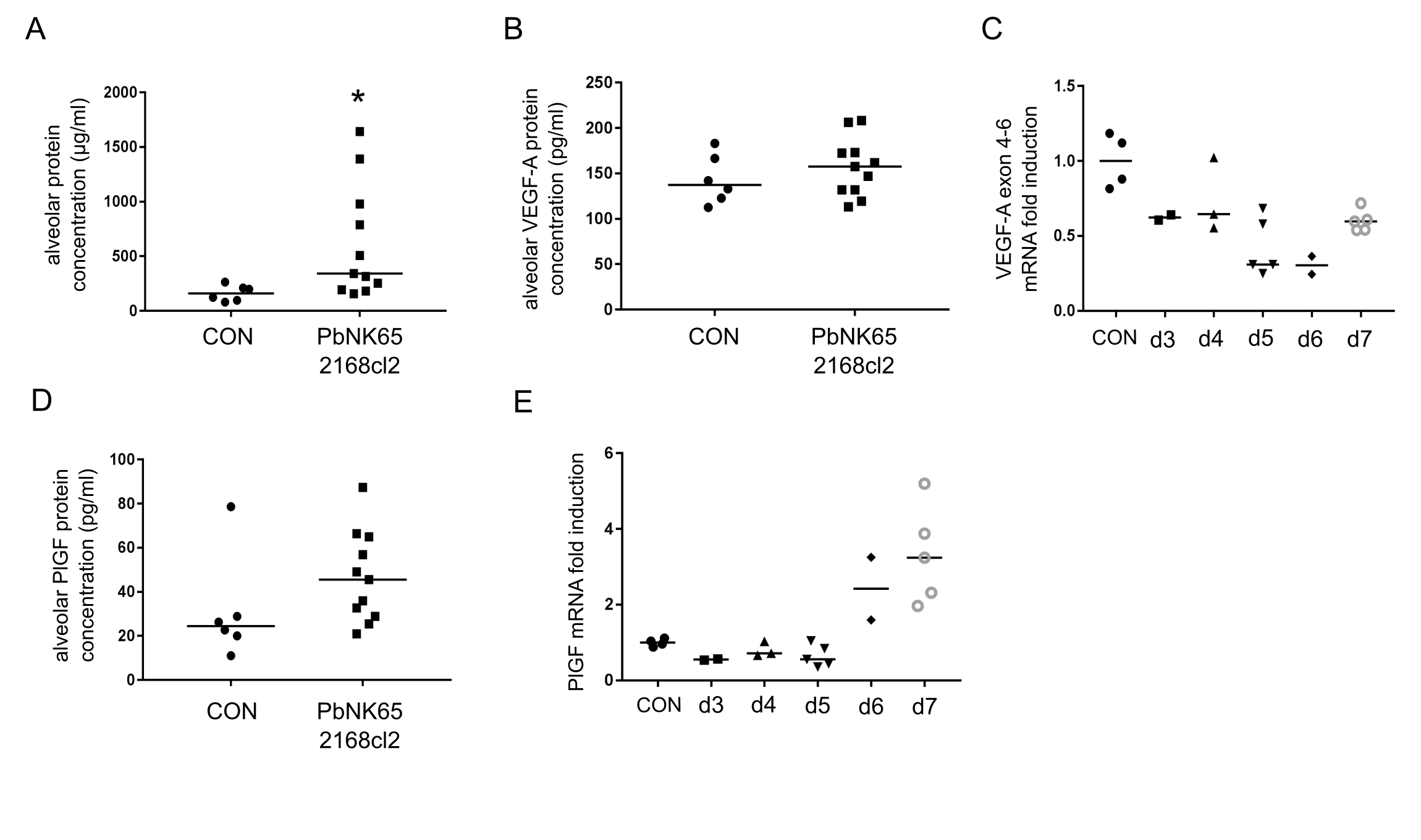

Supplement: Supplementary Figure 2 — Limited lung pathology on day 6 pi in PbNK65 2168cl2-infected C57BL/6 mice. C57BL/6 mice were infected with PbNK65 2168cl2. (A) BALF samples were collected at day 6 pi. After centrifugation, the protein content of the supernatant was determined. (B) The VEGF-A protein content of BALF was measured. (C) Lungs were dissected at indicated days pi. Lungs were further homogenized and the mRNA expression of a VEGF-A isoform (primer VEGF-A exon 4-6) was analyzed by qRT-PCR. (D) The PlGF protein content of BALF, and (E) mRNA expression of PlGF were determined. Compilation of 2 experiments, n = 2–11 per group. [file Image2.TIF]

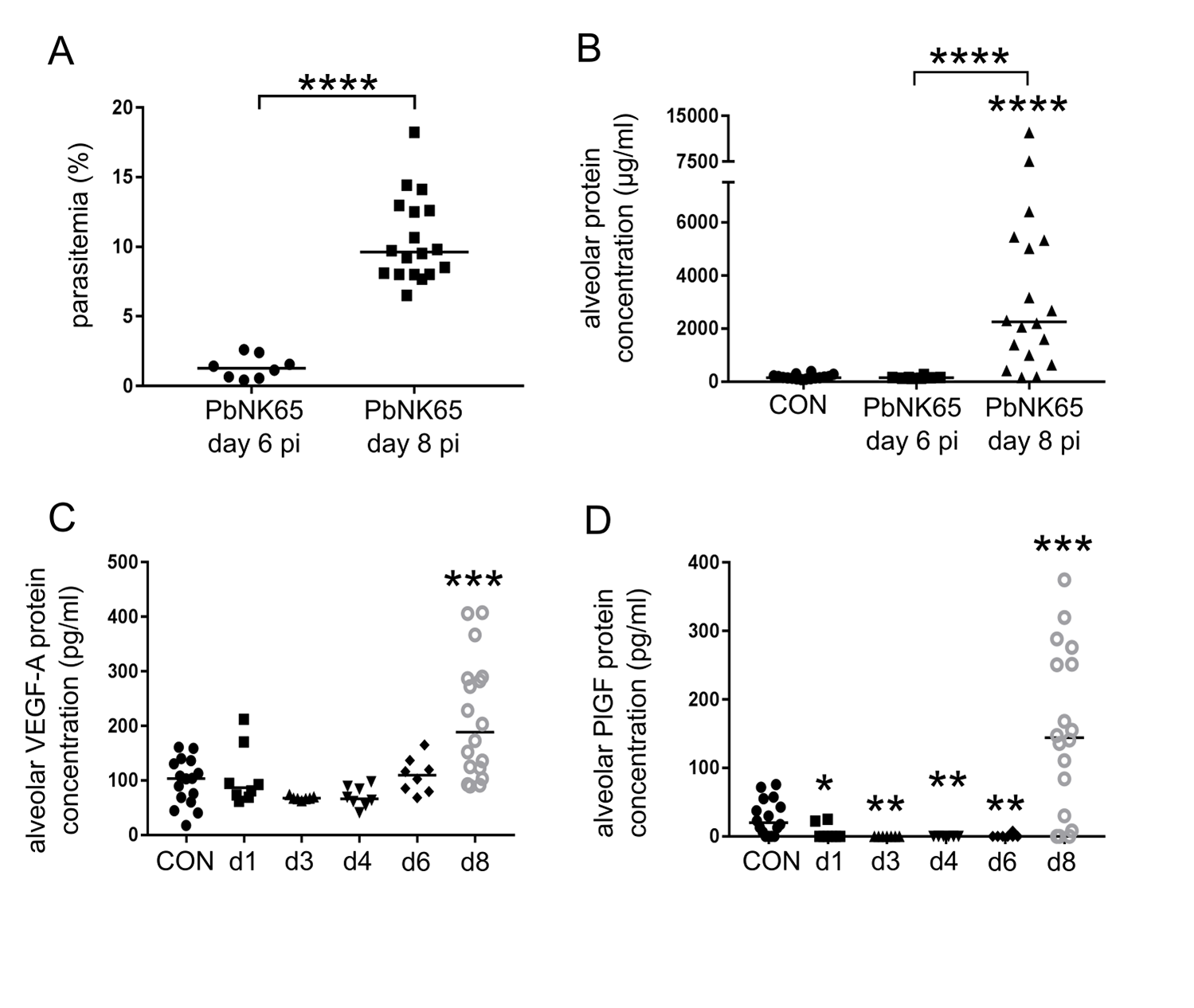

Supplement: Supplementary Figure 3 — Pulmonary VEGF-A and PlGF protein concentrations during PbNK65 infection in C57BL/6 mice. C57BL/6 mice were infected with PbNK65. (A) Peripheral parasitemia was determined at indicated days with Giemsa-stained smears of tail blood and (B) alveolar edema was measured by protein determination in BALF samples. Protein levels of (C,D) VEGF-A and PlGF were also determined in BALF. Compilation of three experiments, n = 7–18 per group. [file Image3.TIF]

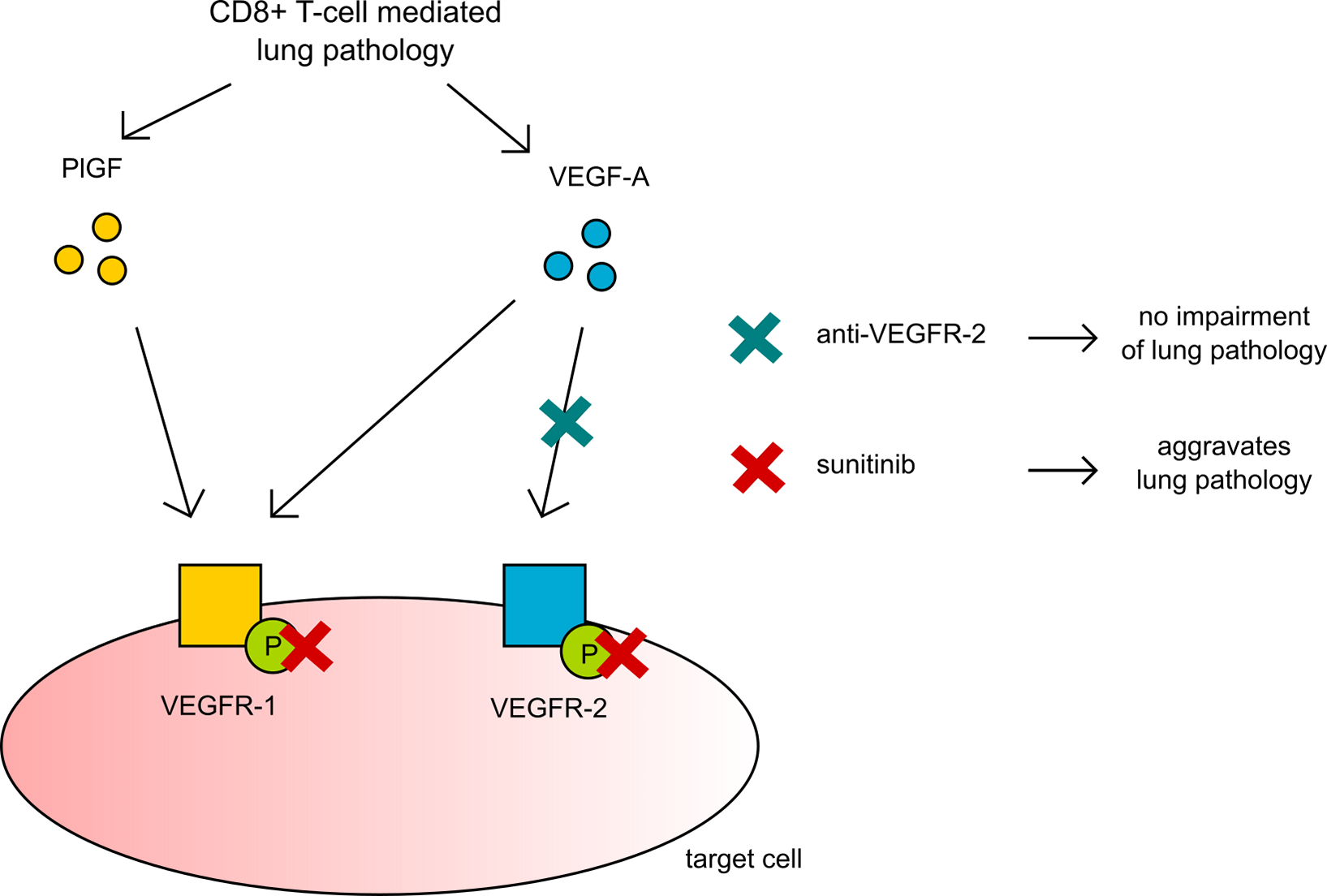

Supplement: Supplementary Figure 4 — Schematic overview of anti-VEGFR-2 and sunitinib treatment in infected mouse lungs. This figure depicts a scheme of the interrelations of the VEGF-A and PlGF pathway with the anti-VEGFR-2 antibody (DC101 clone) and sunitinib treatment in infected mouse lungs. The target cells may be ECs, alveolar type II cells and macrophages. CD8+ T cells were shown to be essential for lung pathology. A downstream effect of this CD8+ T cell-mediated lung pathology is the expression of VEGF-A and PlGF in the lungs, whose receptors are VEGFR-1 and VEGFR-2. The anti-VEGFR-2 antibody inhibits the binding of VEGF-A to VEGFR-2, resulting in decreased VEGFR-2 activation. Sunitinib impairs the tyrosine kinase activity of VEGFR-1 and VEGFR-2 which leads to decreased activation of both receptor tyrosine kinases. However, both treatments did not impair CD8+ T cell-mediated lung pathology and sunitinib even aggravated the pathology. [file Image4.TIF]
